# A Novel Internet of Medical Things Hybrid Model for Cybersecurity Anomaly Detection

**DOI:** 10.3390/s25206501

**Published:** 2025-10-21

**Authors:** Mohammad Zubair Khan, Abdulhakim Sabur, Hamza Ghandorh

**Affiliations:** 1 Department of Computer Science and Information, Applied College, Taibah University, Madinah 42353, Saudi Arabia; mkhanb@taibahu.edu.sa; 2 Energy, Industry, and Advanced Technologies Research Center, Taibah University, Madinah 42353, Saudi Arabia; 3 Department of Cybersecurity, College of Computer Science and Engineering, Taibah University, Madinah 42353, Saudi Arabia; 4 Department of Computer Science, College of Computer Science and Engineering, Taibah University, Madinah 42353, Saudi Arabia; hghandorh@taibahu.edu.sa

**Keywords:** Internet of Medical Things (IoMT) security, anomaly detection, Graph Convolutional Network (GCN), transformer, machine learning

## Abstract

The proliferation of Internet of Medical Things (IoMT) devices connected to the internet poses significant challenges to data integrity, confidentiality, and patient safety due to their vulnerability to outside exploitation. Specifically, IoMT devices capture and process vast amounts of sensitive patient data but often lack adequate security mechanisms, making them susceptible to attacks that compromise data integrity—such as the injection of false or fabricated information—which imposes significant risks on the patient. To address this, we introduce a novel hybrid anomaly detection model combining a Graph Convolutional Network (GCN) with a transformer architecture. The GCN captures the structural relationships within the IoMT data, while the transformer models the sequential dependencies in the anomalies. We evaluate our approach using the novel CICIOMT24 dataset, the first of its kind to emulate real-world IoMT network traffic from over 40 devices and 18 distinct cyberattacks. Compared against several machine learning baselines (including Logistic Regress, Random Forest, and Adaptive Boosting), the hybrid model effectively captures attacks and provides early detection capabilities. This work demonstrates a scalable and robust solution to enhance the safety and security of both IoMT devices and critical patient data.

## 1. Introduction

**Figure 1:**
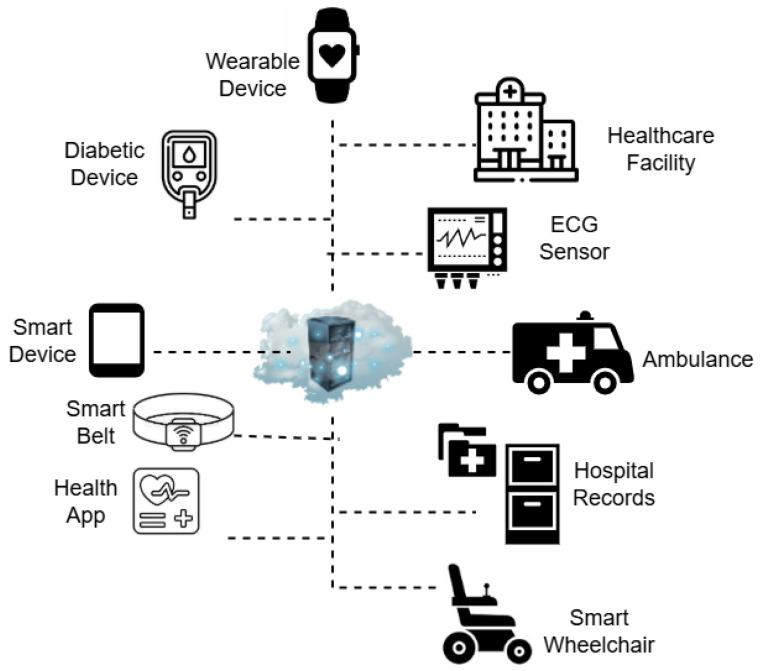
Internet of Medical Things (IoMT) architecture.

IoMT devices are vulnerable to cyberattacks because of the way they are designed, and due to the fact that they are connected to the internet [1]. A typical IoMT architecture has a group of system components, including sensors, gateways, and cloud systems. Also, IoMT systems include a remote monitoring system based on several networking and communication protocols as shown in Figure 1. Hence, the security of the IoMT devices needs to be ensured at various levels since the IoMT devices are handling patients’ data that is both received and sent to different sources. Specifically, IoMT devices are different from other devices because they affect patients’ lives and impose privacy concerns if patients identities are revealed. Attacking the IoMT devices can make the devices stop working, and hence directly affect the patients’ lives.

The detection of cyber attacks on IoMT devices is crucial to prevent the malfunction of the devices and to lower the chance of mistakes that can lead to significant costs such as loss of lives. The detection of cyber attacks using passive methods such as those relying on packet size, packet timestamp, or port numbers are considered ineffective since they do not analyze a network flow and predict the cyber attacks [2]. However, utilizing Machine Learning (ML) models to extract and classify network traffic characteristics has proven to be effective [3,4,5,6]. This challenge is especially evident in early approaches to IoT anomaly detection. Initial methods often depended on handcrafted features and conventional ML models like support vector machines (SVMs) [3]. While these techniques showed reasonable performance in detecting basic anomalies, they fell short when faced with more complex traffic patterns. Their limitations stem from an inability to effectively model the temporal dynamics and structural relationships present in IoT network behavior [7]. While our work also utilizes flow-level features extracted from CICIoMT2024, the proposed GCN–transformer architecture learns higher-order relationships between these features, effectively addressing the representational limitations of prior handcrafted-feature methods.

IoMT devices can also be vulnerable to sensor spoofing attacks [8,9], side-channel inference attacks [10,11,12], and physical injection attacks [13,14]. In sensor spoofing attacks, biometric authentication secures access to sensitive medical devices and patient data. Ni et al. [8] revealed a critical vulnerability in widely adopted in-display fingerprint sensors, demonstrating that these sensors leak fingerprint information via electromagnetic side channels during authentication. Shen et al. [9] introduced FingerFaker, a novel fingerprint spoofing attack that can compromise automated fingerprint recognition systems (AFRSs) without requiring prior knowledge of target fingerprints, addressing a significant limitation of previous spoofing methods that relied on photos and minutiae of the target. Zhang et al. [10] presented a study on OS-level side channel attacks on IOS mobile phones. The authors identified several new side-channel attack vectors like APIs allowing information leakage, and developed machine learning models that increased the accuracy of classifying and detecting those attacks. Jiang et al. [13] introduced WIGHT, the first wired attack capable of creating ghost touches on capacitive touchscreens through charging cables. The research highlights a critical security vulnerability in capacitive touchscreens, which serve as the primary human–machine interface on smart devices. The attack exploits a novel threat vector that only requires a connection to a malicious charging port, such as public charging stations, and remains effective across various power adapters and USB data blockers.

Yet, as network activities become more frequent and diverse, identifying anomalies and attacks against the IoMT devices becomes challenging and difficult due to lack of efficient datasets that include IoMT network traffic [15]. Although IoMT devices are a sub-category of the IoT, their characteristics are unique and they rely on several communication protocols, which adds another layer of difficulty to secure the devices.

In this paper, we present a ML-based approach for IoMT network anomaly detection based on a new dataset published recently in 2024 called CICIoMTD24 dataset [15]. The dataset includes realistic emulations of 40 IoMT devices and 18 different attacks against those devices. Furthermore, the dataset is a multi-protocol and includes Wi-FI, MQTT, and Bluetooth devices. Hence, we can ensure that there is enough diversity in the data and the included features, as we elaborate on later in this paper. The attacks in the dataset are categorized into five classes: DDoS, DoS, MQTT, Reconnaissance, and Spoofing attack. To the best of our knowledge, this dataset is the first of its kind that has realistic emulations of IoMT devices networking traffic with multi-communication protocols. Our approach is to build a strong anomaly detection classifier capable of capturing and distinguishing cyberattacks against IoMT devices. To do this, we first present three ML baseline models, AdaBoost (AdaBoost), Random Forest (RF), and Logistic Regression (LR) [16]. To enhance the detection of anomalies in IoMT networks, we propose a hybrid model, Graph Convolutional Networks (GCN), and a transformer architecture, leveraging the CICIoMTD24 dataset [15]. The GCN component, consisting of two convolutional layers, captures spatial relationships within a k-NN graph constructed from the dataset’s features, while the transformer encoder, equipped with multiple attention heads, effectively models temporal dependencies inherent in IoMT network traffic. By concatenating the outputs of these components and applying a fully connected layer with sigmoid activation, the model performs binary classification to distinguish benign from malicious traffic. This hybrid approach combines the strengths of graph-based learning and self-attention mechanisms, enabling robust handling of the complex, long-range dependencies present in multi-protocol IoMT data. The model’s performance is rigorously evaluated using accuracy, precision, recall, and F1-score, with training dynamics visualized through loss and accuracy curves to ensure a balanced fit.

The contribution of this work is two-folded:

- A novel hybrid ML-based IoMT network anomaly detection that combines transformer–GCN architecture. This hybrid model is able to capture dependencies in the anomaly data of the IoMT devices effectively since the GCN can model the interactions between the data, and the transformer can capture the sequential dependencies of the data.
- A rigorous comparative evaluation of the proposed hybrid transformer–GCN model against traditional ML baselines on the ICICIoMT2024 dataset is conducted. This analysis highlights the hybrid model’s superior ability to capture both structural and temporal dependencies in IoMT anomaly data. The evaluation framework also identifies performance trade-offs and generalization potential, laying the groundwork for future benchmarking across diverse IoMT datasets.

The rest of the article is organized as follows: Section 2 and Section 3 demonstrates the background and related works. Section 4 describes the model details along with the dataset description and the data analysis. Section 5 depicts the performance of the proposed model. Section 6 and Section 7 sheds a light upon the research discussion, conclusion, and future directions.

## 2. Background

This section discusses the foundational concepts and relevant background pertaining to the IoMT.

### 2.1. Internet of Things (IoT) Devices

Internet of Things devices (IoT) have attracted significant attention over recent years due to their cost, deployment, and effectiveness. The number of connected IoT devices in 2024 was 18.8 billion and this is projected to grow to 40 billion by 2030 [17]. One of the main reasons for this growth is the fact that IoT devices are compact, communicate in various communication protocols, and have low power consumption [18,19,20,21,22,23,24]. One of the most widely adopted applications of IoT is its usage in the healthcare sector. Ensuring health is of paramount importance to lead to a successful and content life. Health is defined as the process of maintaining or improving health with the help of diagnosis, prevention, and treatment for illness and injury. To facilitate this, the manual management and maintenance of the patients’ data, history, diagnostics, medication, billing, and drugs, has been adopted by most healthcare providers around the world [25]. Neverthless, IoT has emerged to solve such classical problems of the healthcare sector by overcoming human errors and helping the physician to diagnose diseases more easily and accurately through interconnecting all the vital parameters across different medical devices, which helps in making accurate decisions. Hence, the healthcare community has come up with the term ’Internet of Medical Things’ which refers to medical devices with the facility to transfer data over a network without requiring human-to-human or human-to-computer interaction [25,26,27].

### 2.2. Internet of Medical Things (IoMT)

The Internet of Medical Things (IoMT), a subset of Internet of Things (IoT) technologies, comprises interconnected medical devices used for healthcare monitoring [28]. It integrates interconnected medical devices, sensors, and software applications for autonomous medical data acquisition, transmission, and processing [29].

The significance of the IoMT is evidenced by its capacity to enhance healthcare services through real-time monitoring, cost reduction, and data-driven decision-making [29]. IoMT facilitates the seamless exchange of biomedical data, enabling healthcare providers to deliver precise and personalized care. Furthermore, it supports clinical research by providing access to extensive datasets from interconnected devices, thereby advancing medical science [29]. IoMT is crucial in modern healthcare for its role in remote patient monitoring, personalized treatments, and early detection of health issues, which collectively improve patient outcomes and decrease hospital readmissions [30]. The transformation of traditional healthcare systems by IoMT, particularly in efficiency and treatment standards, is driven by advancements in microprocessors, biosensor architecture, and 5G technologies [16].

The IoMT architecture is typically structured into three distinct layers: the data acquisition layer, the personal server layer, and the medical server layer [31]. Each layer fulfills a specific function essential for IoMT system functionality and security. The data acquisition layer gathers biomedical and contextual signals via various sensor devices. Subsequently, the personal server layer processes and temporarily stores patient data from medical equipment. Finally, the medical server layer, comprising high-performance data centers and cloud servers, facilitates centralized patient control, long-term behavioral analysis, and intelligent decision-making [31].

The widespread implementation of the IoMT is impeded by several challenges. High upfront costs associated with IoMT technology can delay profitability and deter adoption. Significant security concerns arise from the transmission of protected health information, exposing providers to risks such as fraud, data breaches, and credential theft, which may result in severe noncompliance penalties [30]. A critical challenge is the heterogeneity of collected data. Health records from various clinics often contain biases and noise, leading to discrepancies in training AI systems [28]. The diverse nature of IoMT networks, encompassing various devices and protocols, further complicates the implementation of universal security solutions. Additionally, the limited size and battery capacity of these devices restrict their ability to execute cryptographic measures and intensive security computations [16]. Hardware resource limitations in IoMT devices represent another significant challenge. These devices frequently possess constrained computational capacity, memory allocation, and energy efficiency, impeding the implementation of advanced AI algorithms and real-time operations [29]. Furthermore, interoperability poses a substantial hurdle, as IoMT devices from different manufacturers often utilize varying data formats and communication protocols. This lack of standardization complicates seamless integration and data exchange across healthcare systems, thereby limiting the efficiency and effectiveness of IoMT solutions [32].

### 2.3. ML and IoMT

ML, an artificial intelligence method, facilitates predictions through data pattern recognition [28]. ML is categorized into three main types: supervised learning, unsupervised learning, and reinforcement learning [28]. ML is crucial for analyzing extensive datasets, identifying patterns, and generating predictions, rendering it applicable across various fields, including healthcare [16,33]. It significantly enhances the functionality and decision-making capabilities of medical devices within the IoMT [28]. ML algorithms process large volumes of IoMT device data, enabling accurate predictions and real-time health monitoring [28]. Supervised learning, in particular, yields clinically relevant results in healthcare for tasks such as disease prediction, biomarker analysis, and patient monitoring [28].

Ensemble methods are pivotal in bolstering the security of IoMT networks [34]. They combine multipleML models to improve predictive accuracy and robustness, leveraging the strengths of individual base models while mitigating their weaknesses, which facilitates more effective detection of cyber threats [34]. In IoMT contexts, ensemble methods address critical challenges such as data fusion complexity, evolving attack patterns, and resource constraints [35]. Through architectural diversity, ensemble models ensure comprehensive coverage of intrusion scenarios, thereby enhancing detection capabilities across varied datasets and domains [35].

The three primary ML types are supervised, unsupervised, and semi-supervised learning [31,33]. Supervised learning involves training models on labeled data, where inputs are paired with corresponding outputs [31]. This approach is utilized for regression and classification tasks, including predicting continuous values or categorizing data into discrete groups [31]. Common techniques in supervised learning for detecting known attack patterns and classifying medical data include SVM, DT, RF, Naive Bayes (NB), and Artificial Neural Networks (ANN) [31]. Unsupervised learning is applied to unlabeled data, where models identify patterns, relationships, or clusters within the dataset [31]. Techniques like K-means, k-Nearest Neighbor (k-NN), and Self-Organizing Maps (SOM) are employed to group data into meaningful clusters [31]. This method is particularly effective for anomaly detection, flagging deviations from normal behavior as potential threats [31]. Semi-supervised learning integrates both labeled and unlabeled data to improve classification accuracy and minimize the need for extensive manual labeling [31]. This approach is beneficial in scenarios with limited labeled data but abundant unlabeled data, making it suitable for IoMT systems with diverse and dynamic datasets [31].

#### 2.3.1. Logistic Regression (LR)

Logistic regression (LR) is a straightforward and interpretable algorithm predicated on the assumption of linearity between dependent and independent variables [28,36]. While effective for binary classification and providing well-calibrated outputs, its performance diminishes with nonlinear problems and high-dimensional datasets, limiting its utility in complex scenarios [28]. Its inherent simplicity and efficiency are advantageous for direct datasets, yet these qualities constrain its applicability when intricate relationships are present [28].

#### 2.3.2. Random Forest (RF)

Random Forest (RF) is a more robust algorithm that employs multiple decision trees to enhance classification accuracy and manage noisy data [28]. It excels with large and heterogeneous datasets, offering automatic feature selection and requiring no input normalization [28]. However, RF can be computationally expensive, exhibiting slower learning speeds and difficulties in handling missing values [28]. Although susceptible to overfitting if tree depth and quantity are not appropriately defined, its capacity to manage complex data renders it a preferred choice for intricate problems [28].

#### 2.3.3. Adaptive Boosting (AdaBoost)

The Adaptive Boosting (AdaBoost) method constructs a series of weak classifiers, selecting the optimal classifier based on current sample weights after each iteration [37]. AdaBoost offers several advantages: it is a fast, simple, and easy-to-create classification method; it requires minimal configurable parameters; it does not necessitate prior knowledge of the weak learners; it can be integrated with other methods for discovering weak hypotheses; and it consistently generates valid weak hypotheses when sufficient data are provided [37]. Furthermore, it provides a set of theoretical guarantees [37]. However, the model may under perform when data are inadequate, weak hypotheses are overly complicated, or weak hypotheses are too fragile [37].

### 2.4. Graph Conventional Network (GCN)

Graph Neural Networks (GNNs) are DL models designed to operate on graph-structured data, which is composed of nodes (entities) and edges (relationships). Through a process of message passing, GNNs capture the dependencies within a graph to perform tasks such as node classification, link prediction, and graph-level classification.

Various GNN architectures exist, including Graph Convolutional Networks (GCN), Graph Attention Networks (GAT), and Graph Recurrent Networks (GRN). These models leverage graph topology and node features to learn effective representations for applications in domains such as social networks, molecular modeling, and knowledge graphs [38].

GCN is a neural network model developed for semi-supervised learning on graph-structured data. It functions through a layer-wise propagation rule derived from a first-order approximation of spectral graph convolutions, which incorporates the graph’s adjacency matrix and node features. By encoding both local graph structure and node features, GCNs can classify nodes even when labels are available for only a small subset. The model scales linearly with the number of graph edges, ensuring computational efficiency for large graphs [39].

**Figure 2:**
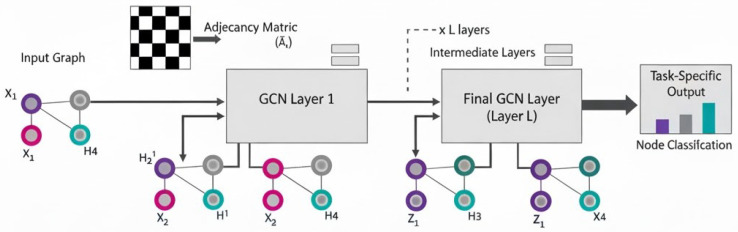
Overview of Graph Convolutional Network (GCN) architecture.

Figure 2 shows an overview of GCN architecture. The GCN takes two main inputs: the initial Input Graph with raw node features ($X_{i}$), and the graph’s structure, represented by the Adjacency Matrix ($\overset{ˇ}{A}$). The model operates by stacking multiple GCN Layers (*L* layers). Each layer performs a graph convolution, updating a node’s feature representation by aggregating and transforming the features of its neighbors. The output of the final layer (*L*) consists of highly contextualized node embeddings ($Z_{i}$), which are then passed to a Task-Specific output layer (Linear and Softmax) to predict the class or category for each individual node.

GCNs are significant for their ability to address key challenges in learning from graph-structured data, which is prevalent in numerous real-world applications. Unlike traditional ML models, GCNs are designed to operate directly on graphs, effectively leveraging both node features and the graph structure to improve representation learning and classification. The formal GCN layer can be defined as follows:

(1) $$H^{\left(l+1\right)}=\sigma \left(\tilde{A}H^{\left(l\right)}W^{\left(l\right)}\right)$$

where $H^{\left(l+1\right)}$ represents the feature matrix at layer l, $\tilde{A}$ is the normalized adjacency matrix, $W^{\left(l\right)}$ is the learnable weight matrix at layer l, and σ is an activation function like ReLU.

A key strength of GCNs is their performance in semi-supervised contexts, where they propagate information from a small subset of labeled nodes across the graph. The model is also computationally efficient, scaling linearly with the number of graph edges, which makes it suitable for large-scale networks such as social, citation, and knowledge graphs.

### 2.5. Transformer

A transformer is a neural network architecture for sequence transduction tasks, such as language modeling and machine translation. It operates entirely on self-attention mechanisms to model dependencies between input and output sequences, thereby eliminating recurrent and convolutional layers. The term “self-attention transformer” refers to the foundational architecture introduced by Vaswani et al. [40], which is characterized by its use of a self-attention mechanism to compute global dependencies between all tokens in an input sequence. This original model, typically applied to sequential data (e.g., BERT, GPT), utilizes positional encodings to preserve token order and may be structured as an encoder–decoder, encoder-only, or decoder-only architecture. Formally, the transformer is the scaled dot-product attention that computes the attention score using the following formula:

(2) $$\mathrm{Attention}\left(Q,K,V\right)=\mathrm{Softmax}\left(\frac{QK^{⊤}}{\sqrt{d_{k}}}\right)V$$

where Q,K, and *V* are the query, key, and value matrices derived from the input sequence, and $d_{k}$ is the dimensionality of the key vectors. The softmax function ensures that the attention weights sum up to one, enabling the model to focus on relevant parts of the sequence. The scaling factor $\sqrt{d_{k}}$ prevents the dot-product values from growing too large, which could cause vanishing gradients during backpropagation.

To improve the model’s capacity to capture complex patterns, the transformer architecture employs multi-head attention. This involves executing several attention mechanisms simultaneously, each producing an output known as a “head”. These heads are concatenated and then linearly transformed using a final weight matrix $W^{O}$. Each head ${\mathrm{head}}_{i}$ is computed by applying the attention function to linearly projected versions of the queries, keys, and values using learnable matrices $W_{i}^{Q}$, $W_{i}^{K}$, and $W_{i}^{V}$, respectively.

(3) $$\mathrm{MultiHead}\left(Q,K,V\right)=\mathrm{Concat}\left({\mathrm{head}}_{1},\ldots ,{\mathrm{head}}_{h}\right)W^{O}$$

In this formulation, Q,K, and *V* are the query, key, and value matrices derived from the input sequence. Each attention head is computed as

$${\mathrm{head}}_{i}=\mathrm{Attention}\left(QW_{i}^{Q},KW_{i}^{K},VW_{i}^{V}\right),$$

where $W_{i}^{Q}$, $W_{i}^{K}$, and $W_{i}^{V}$ are learnable projection matrices for the queries, keys, and values, respectively. The outputs of all heads are concatenated and then transformed by a final weight matrix $W^{O}$ to produce the multi-head attention output.

**Figure 3:**
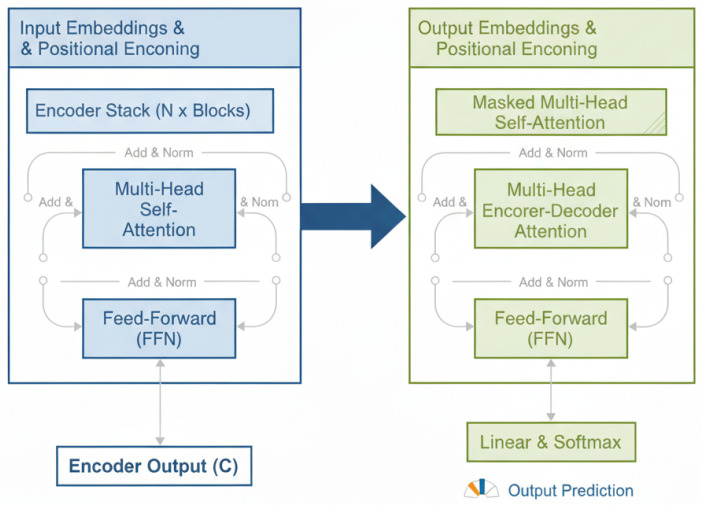
Overview of transformer architecture showing input embeddings and output embeddings stages.

Figure 3 shows an overview of transformer architecture. The image displays the transformer architecture, which is composed of an encoder (left, blue) and a decoder (right, green). The encoder processes input embeddings and positional encodings through a stack of blocks, each containing a Multi-Head Self-Attention layer and a Feed-Forward Network (FFN), using “Add & Norm” for residual connections and normalization to produce a contextualized representation. The decoder receives this representation and uses its own stack of blocks, which includes a Masked Multi-Head Self-Attention layer (to maintain sequence causality), a Multi-Head Encoder–Decoder Attention layer (to focus on relevant input parts), and an FFN, eventually outputting predictions via a final Linear and Softmax layer.

The transformer’s highly parallelizable design allows it to achieve state-of-the-art results in tasks like machine translation, surpassing previous recurrent or convolutional models in both performance and training efficiency [40]. In addition, the model demonstrates significant versatility, with successful applications across diverse modalities including text, images, and graphs [41].

## 3. Related Work

The number of advances in Internet of Things (IoT) security research has risen significantly in recent years. Researchers are looking into the effect of IoT devices on the security of users’ data and how different cyber attacks attempt to cause damage to the IoT devices themselves or to the data. IoMT is a sub-field of IoT with more specialized characteristics of the devices that can create minor or major differences with regular IoT devices [1,22,26,42]. It is estimated that 43% of data breaches in 2021 comprised medical data due to the inefficiency of security solutions protecting this data [26]. Each layer of the IoMT system can be a target of a cyber attack. For example, the perception layer is vulnerable to side channel attacks, the network layer is vulnerable to Denial of Service (DoS) attacks, the session layer is vulnerable to XSS attacks, and the business layer is vulnerable to deception attacks [1,22,26,42,43].

In this section, we present related work on IoMT anomaly detection with a focus on some of the recent works in this domain. We provide a summary of the surveyed papers in Table 1. Our selection followed a systematic, thematic, and methodological criterion focusing on recent (2021–2025) peer-reviewed studies addressing anomaly detection in IoMT systems using ML or DL, with emphasis on multi-class classification. The inclusion criteria were as follows: (1) studies explicitly targeting IoMT or closely related IoT security contexts; (2) proposed models evaluated on IoMT or IoT benchmark datasets; and (3) papers emphasizing network or system-level anomaly detection. Studies focusing purely on general IoT or industrial IoT were excluded unless they introduced architectures (e.g., GCN, transformers) relevant for potential adaptation to IoMT domains.

Authors in [44] proposed a system that integrates IoMT devices to track users actions and interactions with the devices. The system is built using Raspberry Pi devices with the aim of implementing an anomaly-based intrusion detection system (AIDS). The authors employ a local detection engine (LDE) running on IoMT devices and a central detection engine (CDE) running on the backend server/cloud. Several ML and DL methods were tested and evaluated to detect known and unknown attacks against the IoMT devices. The authors used models such as RF, One Class SVM (OCSVM), Outlier detection with Local Outlier Factor (LOF), and k-NN models. The evaluation results indicate that almost all novelty detection algorithms can generally identify both normal and abnormal records related to the network traffic passing through the IoT/IoMT Gateway with high accuracy.

Sasi et al. [45] proposed a 1D-CNN-LSTM self attention system that can detect network anomalies in IoT devices. The proposed mechanism achieves high accuracy and efficiently differentiates malicious and benign network traffic. The model utilizes the Shapley additive explanations (SHAP), identified important predictive features from the preprocessed data, which were retrieved using CICFlowMeter (https://github.com/ahlashkari/CICFlowMeter (accessed on 18 November 2022)). The proposed approach achieves a 98% accuracy of detecting the attacks based on 11 features extracted from the utilized dataset.

Zachos et al. [46] presented an anomaly-based intrusion detection system for the IoMT devices, where they proposed a system that combines techniques from hosts, networks, gateways, and IoMT network edges to collect multiple logs from the devices and applies several ML algorithms to evaluate the proposed system for detecting anomalies in the IoMT. The authors utilized decision trees, Naive Bayes, linear regression, RF, k-nearest neighbor (k-NN), and support vector machine (SVM) as the base ML models to evaluate the proposed approach. The authors utilized the LWSNDR dataset [47] Bot-IoT [48], a network traffic classification dataset [49], a DoS attack on IoT devices dataset [50], the ToN dataset [51], and the behavioral IoT dataset designed for general purpose IoT devices [52].

An unsupervised anomaly detection approach is presented by [53] and is based on clustering and online learning. The authors utilized One Class SVM (OCSVM), Local Outlier Factor (LOF), Elliptic Envelope, and Isolation Forest as the ML models. Authors address the dynamic nature of IoMT data by utilizing online learning, where new data arriving is stored, and for every 5000 samples, the clustering model is reapplied to the augmented dataset. The base dataset contains 50,000 samples with over 25,000 anomaly samples. The obtained results from the evaluated models shows that the LOF model achieved best detection accuracy of 86% aamong all four models.

**Table 1:** Summary of anomaly detection methods in IoMT.

Work	Method/Approach	Limitation
Zachos et al. [44]	Raspberry PI-based system tracking users’ activity using IoMT with anomaly intrusion detection system (AIDS). RF, One Class SVM, LOF, and k-NN models are tested on locally generated data from the Raspberry PI devices.	Unrealistic dataset in terms of limited IoMT device type and low detection accuracy on the used ML models.
Sasi et al. [45]	1D-CNN-LSTM self-attention system for anomaly detection in IoT devices.	Limited set of features. The model is not designed for IoMT, which differs from the general IoT devices.
Ea et al. [53]	Unsupervised model based on OCSVM with LoF and IF model.	Low accuracy and the utilized dataset is not designed for IoMT.
Kirubavathi et al. [54]	AutoEncoder and TabNet models	The model tends to overlook some attack instances, resulting in lower recall for malicious activities.
Reji et al. [55]	RF, DT, LR, SVM, and k-NN.	The evaluation dataset is limited in terms of the included attacks, and it is not designed for IoMT.
Torre et al. [56]	Federated learning using 1D-CNN.	Low anomaly detection accuracy in comparison with other state-of-the-art models. Also, the chosen dataset is not designed specifically for IoMT, but for general IoT devices.
Gao et al. [57]	Graph convolution + transformer.	Focused on industrial IoT; does not address IoMT-specific requirements such as patient safety, device interoperability, and healthcare data regulations.
Yang et al. [58]	Sparse Autoencoder + Graph Transformer network for unsupervised anomaly detection in CPS/industrial IoT.	Designed for industrial CPS; not tailored to IoMT-specific needs such as patient safety, clinical data privacy, or medical device interoperability.

The paper [54] proposes a hybrid anomaly detection framework for IoMT systems that combines AutoEncoder and TabNet models. AutoEncoders effectively identify benign traffic with high precision but tend to overlook some attack instances, resulting in lower recall for malicious activities. Conversely, TabNet leverages attention mechanisms to improve recall in balanced datasets but struggles with highly imbalanced and unseen attack patterns. By integrating these models, the framework achieves a balanced trade-off between precision and recall, providing a scalable and adaptable solution for real-time cybersecurity in heterogeneous IoMT environments.

Reji et al. [55] propose a ML-based anomaly detection system for the IoMT to enhance cybersecurity against cyber threats. The authors implement and evaluate various algorithms, including RF, DT, LR, support vector machine (SVM), and k-NN, using the publicly available TON IoT dataset, which simulates multiple attack scenarios such as scanning, DoS, password cracking, and MITM. Their results indicate that SVM models generally outperform other algorithms in detecting network anomalies related to IoMT devices, demonstrating high accuracy and low false positive rates. The study emphasizes the importance of efficient feature selection to reduce computational costs, achieving significant savings in training time with minimal impact on performance.

Torre et al. [56] developed a Federated Learning (FL) Intrusion Detection System (IDS) using a 1D Convolutional Neural Network (CNN) for IoT networks. The CNN architecture includes an input layer, a 1D convolutional layer (32 filters, kernel size 3, ReLU activation), a max pooling layer (pool size 2), a flattening layer, a dense layer with 128 units (ReLU), a dropout layer (rate 0.5), a batch normalization layer, and a softmax output layer. For privacy preservation, three mechanisms were applied to the model parameters: Differential Privacy (noise added using a Laplace Gamma distribution), Diffie–Hellman Key Exchange (secure offset encryption), and Fully Homomorphic Encryption (randomized operations on encrypted weights). The model was tested on seven IoT datasets (TON-IoT, IoT-23, BoT-IoT, CIC IoT 2023, CIC IoMT 2024, RT-IoT 2022, and EdgeIIoT), achieving an average accuracy of 97.31%, precision of 95.59%, recall of 92.43%, and F1-score of 92.69%. Privacy mechanisms introduced a 10% computational overhead compared to non-encrypted FL.

Recent research on multivariate time series anomaly detection in IoT systems has explored both statistical and ML approaches, but many struggle to capture the nonlinear spatial-temporal dependencies among sensors. DL models such as RNNs, GANs, and AutoEncoders have improved temporal modeling, while graph neural networks (GNNs) have been introduced to better represent sensor interconnections. However, most GNN-based methods assume fixed or sparsely constructed graph structures, limiting adaptability to dynamic environments. Similarly, transformer-based models enhance long-term temporal modeling but often lose positional order due to the self-attention mechanism. To address these challenges, Gao et al. [57] propose a Dynamic Deep Graph Convolution with Enhanced Transformer (DDGCT), which jointly learns dynamic sensor graph structures and employs a patch-based transformer with relative position encoding to capture both spatial and temporal dependencies, achieving state-of-the-art anomaly detection in industrial IoT datasets. While highly relevant to industrial applications, this work is not specifically tailored to IoMT scenarios, as it does not address domain-specific issues such as patient-centered safety, medical device interoperability, or compliance with healthcare data protection standards. Yang et al. [58] introduce SGTrans, a framework for multivariate time series anomaly detection in cyber–physical systems. The method integrates a Sparse Autoencoder for dimensionality reduction with a Graph Transformer network that employs multi-head attention to model long-range spatiotemporal dependencies. By combining reconstruction and forecasting errors, SGTrans achieves superior detection performance across benchmark industrial datasets, including SWaT, WADI, and SMAP.

The reviewed studies primarily address IoMT anomaly detection using either traditionalML, DL, or graph-based approaches, often focusing on temporal patterns or static graph structures separately. However, they tend to overlook the combined exploitation of both spatial relationships in IoMT networks and long-range temporal dependencies, as well as the integration of lightweight architectures suitable for real-time applications. In contrast, the proposed work uniquely fuses a Graph Convolutional Network with a transformer model to capture complex spatial-temporal correlations while maintaining computational efficiency, thereby offering a more comprehensive and practical solution for real-time anomaly detection in IoMT environments.

## 4. Experimental Design, Materials and Methods

### 4.1. Multi-Protocol Dataset for Assessing IoMT Device Security (CICIoMT2024)

**Figure 4:**
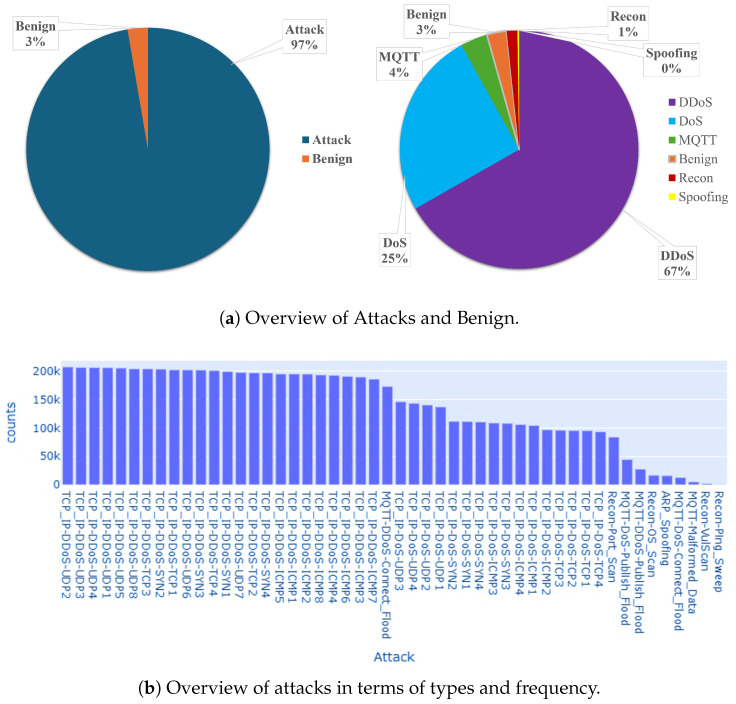
Distribution of the CICIoMT2024 dataset.

Figure 4 shows an overview of the CICIoMT2024 dataset. The dataset is curated by Dadkhah et al. [59] and acts as a realistic benchmark dataset for developing and evaluating IoMT security solutions. Through the data collection process, 18 attacks were executed on an IoMT testbed consisting of 40 devices, including 25 real and 15 simulated attacks. Furthermore, the dataset includes multiple healthcare protocols such as Wi-Fi, MQTT, and Bluetooth. The total counts of network traffic attacks is 7,160,831, where 6,968,099 are classified as attacks and 192,732 as benign.

The dataset has a variety of features extracted from the collected network traffic. For instance, ‘Header Length’ (mean transport layer header lengths), Time-To-Live, Rate (packet transmission speed in packets/s), and proportions of packets with specific flags (FIN, SYN, RST, PSH, ACK, ECE, CWR). It also lists counts of flag occurrences (SYN, ACK, FIN, RST) and average packet numbers per protocol (IGMP, HTTPS, HTTP, Telnet, DNS, SMTP, SSH, IRC, TCP, UDP, DHCP, ARP, ICMP, IPv, LLC). Additionally, it provides statistical measures like *Tot Sum* (total packet length), *Min* (shortest packet), *Max* (longest packet), *AVG* (mean packet length), *Std* (standard deviation), *Tot Size* (average packet length), *IAT* (mean inter-arrival time), *Number* (total packets in the window), *Variance* (packet length variance), and *Protocol Type* (mode of protocols in the window).

It is important to note that the CICIoMT2024 dataset contains flow-level features that are manually engineered from captured packet statistics. While we rely in our work on those predefined features, the learning process is entirely different from traditional handcrafted-feature models. In our approach, we construct a graph from these feature vectors, enabling the GCN component to learn spatial correlations among network flows, while the transformer encoder captures temporal dependencies across sequences of traffic behaviors. The combination between the GCN and the transformer allows the model to move beyond static feature reliance and extract deeper contextual relationships within the IoMT data. The attacks are classified into five categories: DDoS, DoS, Recon, MQTT, and spoofing, where Benign was detected 230,339 times, while DDoS was detected 4,779,859 times, DoS was detected 1,805,529 times, MQTT was detected 262,938 times, Recon was detected 103,726 times, and Spoofing was detected 16,047 times. The attacks details are presented in Table 2.

Attacks were simulated across three protocols—Wi-Fi, MQTT, and Bluetooth Low Energy (BLE)—to emulate real-world IoMT cybersecurity threats. For Wi-Fi, attacks targeting healthcare devices and cameras connected via access points included ARP spoofing, reconnaissance (e.g., port scans), and flooding-based Denial of Service (DoS)/Distributed DoS (DDoS) attacks, such as ICMP, SYN, TCP, and UDP floods. MQTT attacks were executed using the IoTFlock simulation platform and included MQTT Connect Floods, Publish Floods (DoS/DDoS), and Malformed Data attacks, which disrupted broker and subscriber communication Bluetooth (BLE) attacks that involved the continuous writing of data to device characteristics using the Bleak library, with the aim of overloading or disrupting device operations. These BLE tests were conducted inside a Faraday cage to ensure signal isolation. The generation of malicious traffic focused on prevalent attack types to assess system vulnerabilities and to collect high-quality data for IoMT security research.

### 4.2. Proposed Approach

**Figure 5:**
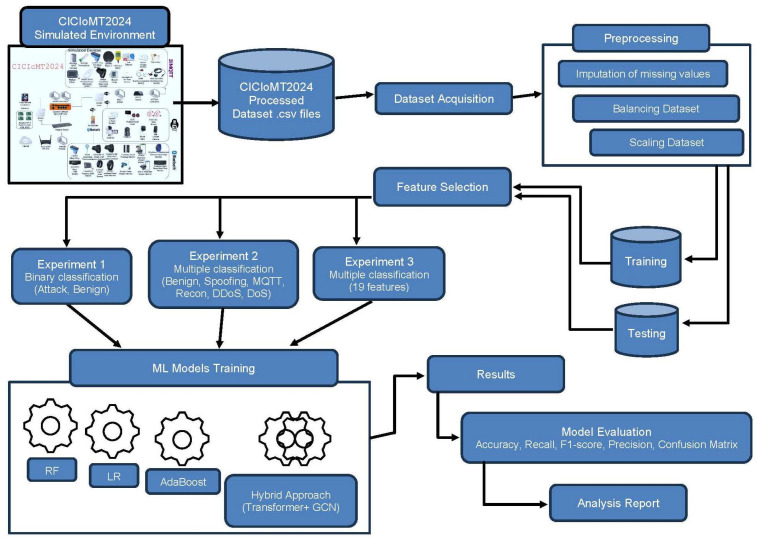
Overview of the proposed approach and its four phases. The proposed approach consists of four phases: data collection, data preprocessing, model building, and model performance evaluation.

Figure 5 illustrates the proposed approach to carry out the research. The following sections describe each phase in more detail.

The model follows a structured ML pipeline to classify network traffic data. It begins with the data preprocessing stage, where missing values are replaced, numerical features are normalized using MinMaxScaler, and categorical labels are encoded for ML compatibility. To address class imbalance, SMOTE is applied, ensuring equal representation of all classes. Next, in the model selection and initialization stage, multiple algorithms, including LR, AdaBoost, RF, and transformer–GCN, are chosen and trained. Feature selection is performed using Decision Tree (DT) importance to identify the top features contributing to classification. Each model is evaluated using metrics like accuracy, precision, recall, F1-score, and ROC AUC, with confusion matrices and visualizations providing deeper insights into their performance. RF emerges as the best-performing model, achieving near-perfect accuracy and robustness in predictions.

The proposed model leverages several powerful libraries and tools to streamline the ML pipeline. Python 3.8 libraries such as pandas and ‘numpy’ are used for data manipulation and numerical computations. Visualization tools like ‘matplotlib’ and ‘seaborn’ are employed to create plots and heatmaps for data exploration and evaluation. ML libraries, including scikit-learn and ‘imblearn’, provide essential functions for preprocessing, model training, and evaluation. TensorFlow and Keras are utilized for building and training the DL model. These tools collectively enable efficient implementation and analysis of the ML pipeline.

Although the CICIoMT2024 dataset is represented as tabular flow records, the underlying IoMT traffic exhibits intrinsic relational and sequential dependencies that cannot be fully captured by flat feature-based models. Each record originates from device-level interactions governed by shared communication protocols and temporal co-occurrence in network sessions. To model these relationships, we construct a k-nearest neighbor (k-NN) graph from the feature space, where nodes represent individual traffic flows and edges encode similarity among their statistical or protocol attributes. The GCN component learns structural dependencies across these correlated flows, enabling the model to capture contextual anomalies that traditional classifiers overlook. The transformer encoder complements this by modeling temporal dependencies between consecutive flows within each session, capturing evolving attack signatures and burst-like behaviors that often precede IoMT intrusions. This hybrid design therefore, integrates spatial correlation (via GCN) with temporal dynamics (via transformer), providing a theoretically grounded framework tailored to the heterogeneous and time-varying nature of IoMT network data.

### 4.3. Data Preprocessing

The proposed model begins with a comprehensive data preprocessing stage to ensure the dataset is clean and ready for analysis. Missing values are handled by replacing them with the mode of the respective columns, ensuring no gaps in the data that could hinder model performance. The dataset contains both numerical and categorical features, which are treated differently. Numerical features are normalized using ‘MinMaxScaler’ to scale values between 0 and 1, making them suitable for ML algorithms. Categorical features, such as labels, are encoded using techniques like ‘OrdinalEncoder’ to convert them into numerical representations. This step ensures that all features are in a format compatible with ML models.

### 4.4. Model Selection and Initialization

The proposed model employs a diverse set of ML algorithms to classify network traffic data effectively. LR, AdaBoost, RF, and a DL model are selected for training and evaluation. Each algorithm is initialized with appropriate hyperparameters to optimize performance. LR is configured for multi-class classification, AdaBoost is used for boosting weak learners, and RF leverages ensemble learning for robust predictions. The DL model is built using TensorFlow and Keras, featuring multiple dense layers and dropout for regularization. This variety of models ensures a comprehensive comparison of performance across different approaches.

### 4.5. Data Normalization and Scaling

To ensure the features are on a comparable scale, the proposed model applies normalization and scaling techniques. Numerical features are normalized using ‘MinMaxScaler’, which transforms values to a range between 0 and 1. This step is crucial for algorithms sensitive to feature magnitudes, such as LR and DL models. Scaling ensures that no single feature dominates the learning process, improving the model’s ability to generalize across different data points. By normalizing the data, the model achieves better convergence during training and avoids issues related to feature disparity.

**Figure 6:**
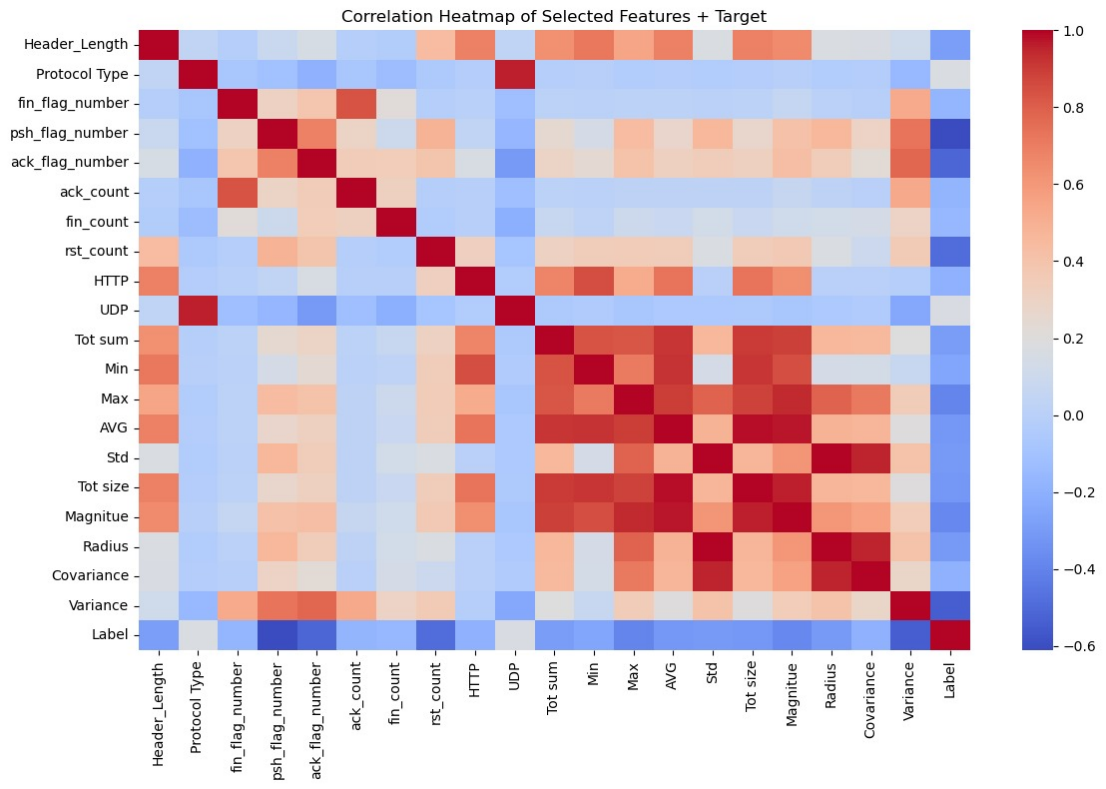
Correlation heatmap between the selected features of the CICIoMT24 dataset. The color intensity signifies the strength of the correlation, where red indicates a positive correlation, blue indicates a negative correlation, and gray indicates a weak correlation.

Figure 6 summarizes the linear relationships between the dataset’s features. Strong positive correlations exist between ‘AVG’ and both ‘Magnitude’ and ‘Tot size’. A significant positive correlation is also observed between ‘Protocol Type’ and ‘UDP’. Additionally, ‘Covariance’ shows strong positive correlations with both ‘Radius’ and ‘Std’. Also, ‘Radius’ and ‘Std’ show strong positive correlations. In contrast, significant negative correlations are observed between the ‘Label’ and several other features, including ‘psh_flag_number’, ‘ack_flag_number’, ‘rst_count’, and ‘Variance’. Finally, the heatmap indicates a lack of correlation for certain features. For instance, ‘HTTP’ and ‘ack_flag_number’ appear largely independent of other variables. Similarly, ‘Header_Length’ shows little to no correlation with ‘Radius’, ‘Covariance’, ‘Variance’, and ‘ack_flag_number’.

### 4.6. Data Splitting and Balancing

The dataset is split into training and testing sets to evaluate the model’s performance on unseen data. The proposed model uses an 80-20 split, ensuring that 80% of the data is used for training and 20% for testing. Stratified sampling is applied to maintain the class distribution across both sets, preventing bias in the evaluation process. This step is critical for assessing the model’s ability to generalize and perform well on real-world data. The training set is used to fit the model, while the testing set provides an unbiased evaluation of its predictive capabilities.

**Figure 7:**
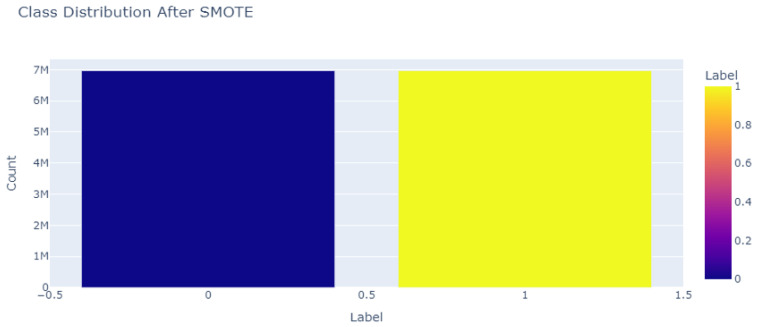
CICIOMT24 Class distribution after applying SMOTE. After applying the SMOTE technique, the dataset was balanced, with both the ’Benign’ (label 0) and ’attack’ (label 1) classes now containing 6,968,099 samples each.

To address the issue of class imbalance, the proposed model employs SMOTE (Synthetic Minority Over-sampling Technique [60]). SMOTE generates synthetic samples for minority classes, ensuring equal representation of all classes in the dataset. This step is essential for preventing the model from being biased toward majority classes and improving its ability to classify minority classes accurately. After applying SMOTE on the training dataset, a balanced distribution was achieved, enabling fair training and evaluation of the model across all classes. We show balanced data after applying the SMOTE to obtain an equal data distribution in Figure 7.

### 4.7. Proposed Architecture and Model

**Figure 8:**
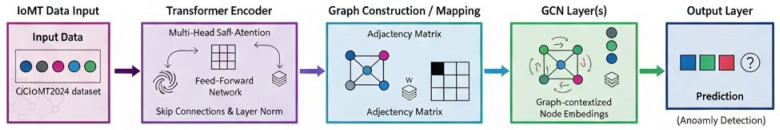
Overview of the hybrid transformer–GCN architecture.

From Figure 8, an overview of the hybrid transformer–GCN model architecture is presented. The model combines a transformer encoder with a GCN where it aims at anomaly detection and attacks classifications. First, the time series input is processed by the transformer encoder (i.e., using Multi-Head Self-Attention and Feed-Forward Network) to capture long-range dependencies and generate contextualized features. Next, these features are mapped into a structured graph defined by an Adjacency Matrix (W), explicitly modeling the relationships between the features. Finally, the GCN Layer(s) aggregate information across the graph’s connections, producing graph-contextualized Node Embeddings that are passed to the output layer to generate the final prediction.

### 4.8. Experiments Setting

The key hardware components used for our experiments are as follows:

- CPU: Intel Core i7-10750H, 3.90 GHz (Intel, Santa Clara, CA, USA)
- GPU: NVIDIA GForce GTX 1050 Ti (NVIDIA, Santa Clara, CA, USA)
- RAM: 32GB
- ML/AI: PyTorch V2.8.0 using torch-geometric library
- Hyperparameters: Provided in Table 3.

### 4.9. Feature Selection

Feature selection is performed to identify the most important features contributing to classification. The proposed model uses DT feature importance to rank features based on their relevance. The top 10 features are selected for training, reducing dimensionality and improving computational efficiency. This step ensures that the model focuses on the most informative features, enhancing its predictive accuracy. By eliminating irrelevant features, the model achieves faster training times and better generalization.

**Figure 9:**
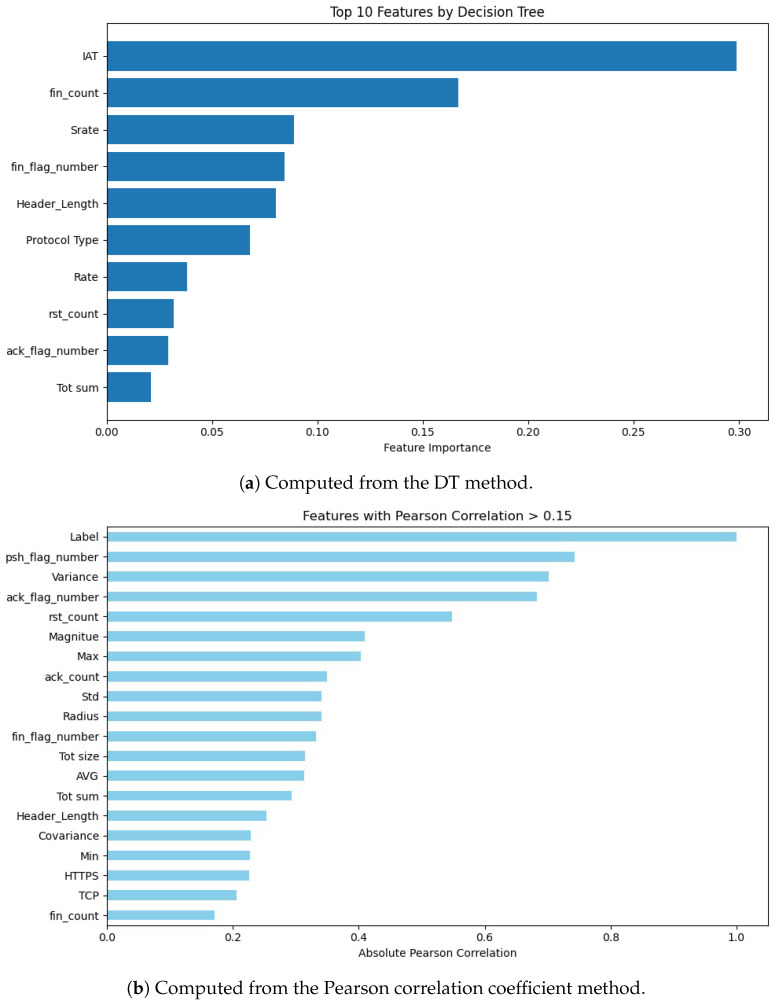
Feature importance for the CICIoMT2024 dataset, computed using the following: (**a**) DT method and (**b**) Pearson correlation coefficient method.

The feature importance for the CICIoMT2024 dataset is shown for DT and the Pearson correlation coefficient methods in Figure 9. From Figure 9a, the DT method identified (‘IAT’, ‘fin_count’, ‘Srate’, ‘fin_flag_number’, ‘Header_Length’, ‘Protocol Type’, ‘Rate’, ‘rst_count’,‘ack_flag_number’, ‘Tot sum’). ‘IAT’ is the most important feature among all the dependent features, while ‘Tot sum’ is the least important feature of the dataset. From Figure 9b, the Pearson correlation coefficient method identified ‘Header_Length’, ‘Duration’, ‘psh_flag_number’, ‘ack_flag_number’, ‘rst_count’, ‘HTTPS’, ‘TCP’, ‘ARP’, ‘IPv’, ‘LLC’, ‘Tot sum’, ‘Min’, ‘Max’, ‘AVG’, ‘Std’, ‘Tot size’, ‘Magnitue’, ‘Radius’, and ‘Variance’. ‘Label’ is the most important feature among all the dependent features, while ‘fin_count’ is the least important feature of the dataset.

### 4.10. Model Selection and Initialization

The proposed study employs a diverse set of models to classify IoMT network traffic data, including LR, AdaBoost, RF (the baseline models), and a hybrid transformer encoder and Graph Convolutional Network (transformer–GCN). LR is configured for multi-class classification, AdaBoost is used to boost weak learners, and RF leverages ensemble learning for robust predictions.

The baseline models were implemented using scikit-learn. LR is configured with a maximum of 1000 iterations to ensure convergence for all datasets. In contrast, AdaBoost uses default parameters to boost weak decision tree learners, and RF is initialized with default hyperparameters, providing an ensemble of decision trees for robust predictions. All models are trained on the balanced dataset obtained after applying SMOTE, and evaluated on unseen test data using accuracy, precision, recall, F1-score, and ROC AUC metrics. For binary classification, the False Positive Rate is also reported. Training times and system resource utilization (CPU and memory) are monitored to provide practical insights into the computational cost of each model. ROC curves are generated per class for multi-class experiments to assess the models’ discrimination capabilities across different attack categories.

The hybrid transformer–GCN model is designed to capture both structural and sequential dependencies in IoMT traffic. It consists of two GCN layers with 64 hidden units and a transformer encoder with two layers, four attention heads, and a projection dimension of 48. Outputs from both components are concatenated and passed through a linear layer with a sigmoid activation for binary classification. Graph edges are constructed via a k-nearest neighbors approach, and the model is trained using the Adam optimizer with a learning rate of 0.001 for 10 epochs, employing Binary Cross-Entropy Loss. Finally, we evaluate the model on unseen test data using accuracy, precision, recall, and F1-score, and ensure that the training and test accuracy curves are monitored for overfitting.

### 4.11. Experimental Setup

To explore how different models respond to increasing complexity, we designed a series of three experiments, each building on the last. We began with a straightforward binary classification task: distinguishing benign traffic from malicious. From there, we moved into more nuanced territory, testing whether models could identify broader attack families. Finally, we pushed the models to their limits by asking them to differentiate between all 18 individual attack types found in the CICIoMT2024 dataset. This progressive setup was not just about measuring performance; it was about understanding how well each model adapts as the classification challenge becomes more complex.

#### 4.11.1. Experiment 1

The first experiment focused on binary classification: separating benign traffic from attacks. We evaluated four baseline models introduced earlier: LR, RF, AdaBoost, and our proposed transformer–GCN hybrid model. This initial test served as a benchmark, helping us gauge how well traditional and hybrid approaches handle basic separation tasks.

#### 4.11.2. Experiment 2

Next, we move on to a coarse-grained anomaly detection task. Instead of isolating individual attack types, we grouped them into five broader categories: Spoofing, Reconnaissance, DDoS, DoS, and MQTT. Subtypes like DoS_SYN were merged into their parent groups (see Table 2). The goal here was to assess whether the models could generalize across attack families while still maintaining meaningful distinctions between them.

#### 4.11.3. Experiment 3

The final experiment introduced the most complex challenge: fine-grained classification across all 18 attack types in the CICIoMT2024 dataset, plus benign traffic. Each model was trained to detect anomalies at the sub-class level, and we evaluated their performance using accuracy, precision, recall, F1-score, ROC AUC, and false positive rate.

### 4.12. Performance Metrics

In our evaluation, we use the following metrics to evaluate each ML model:

- ***Accuracy:*** The accuracy metric evaluate the classification model by calculating the proportion of correct predictions in a dataset by all the samples as follows:
  (4) $$Acc\;=\;\frac{TP+TN}{TP+TN+FP+FN}$$
- ***Recall:*** Recall is defined as the ratio of classes identified to the total number of occurrences of this particular class:
  (5) $$Rec\;=\;\frac{TP}{TP+FN}$$
- ***Precision:*** Precision is defined as the ratio of correctly classified classes to the total number of positive classifications:
  (6) $$Pre\;=\;\frac{TP}{TP+FP}$$
- ***F1-Score:*** The F1 measures the average of precision and recall as follows:
  (7) $$F1\;=\;2\;\times \;\frac{Pre\times \;Rec}{Pre\;+\;Rec}$$

## 5. Results

This section indicates an overview of the three experiments in which the performance of LR, AdaBoost, and RF, were measured. Table 4 illustrated a comparison of the three experiments performance metrics including accuracy, precision, recall, and F1-score.

### 5.1. Experiment 1 Results

**Figure 10:**
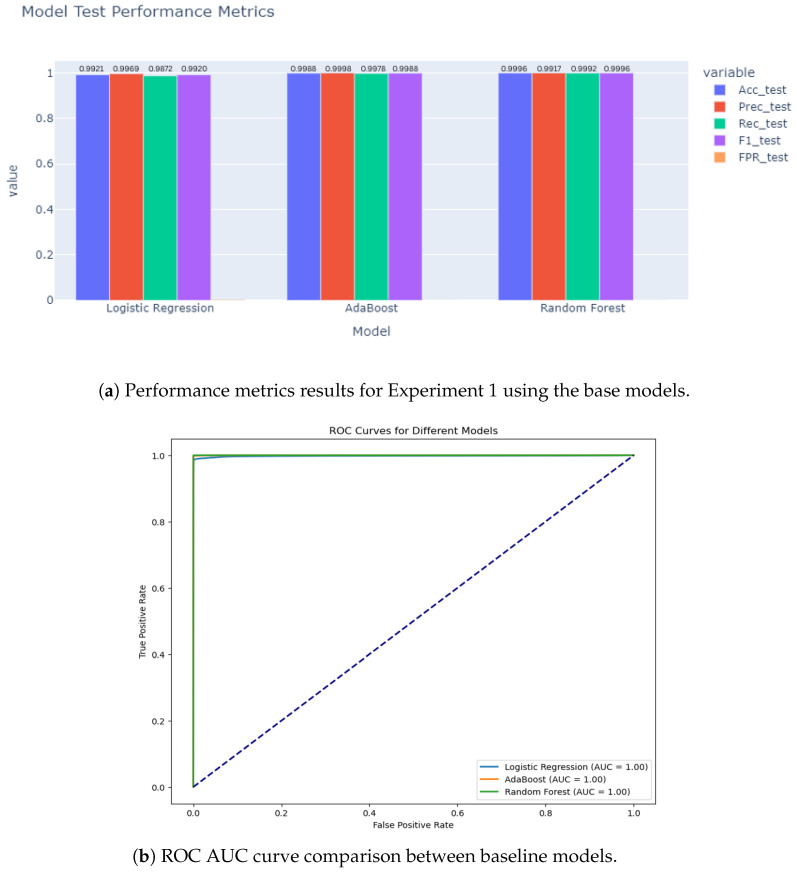
Results from Experiment 1. (**a**) The accuracy, precision, recall, and F1-Score. (**b**) ROC AUC curves for the chosen baseline models.

**Figure 11:**
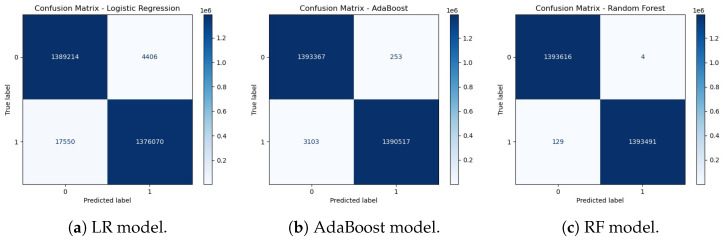
Confusion matrices for LR, AdaBoost, and RF models in Experiment 1.

For the binary classification experiment, we show the comparison between the LR, RF, and AdaBoost models results as shown in Figure 10a. The ROC AUC curve for the experiment is shown in Figure 10b, which also confirms the high detection results achieved by the classification baseline models. The confusion matrices for the first experiment are shown in Figure 11. All three models achieved high detection accuracy for the anomalies in the CICIoMT24 dataset, yet RF was the best with an accuracy of 99.8%, while the LR and AdaBoost were 99.21% and 99.89%, respectively. The exceptionally high performance of the RF and AdaBoost models prompts consideration of potential overfitting. Nevertheless, the use of a sufficiently large dataset and evaluation on previously unseen test data mitigates this concern, suggesting the results are not merely due to memorization. The experiment was framed as a binary classification problem, distinguishing between normal and anomalous IoMT traffic. Within this context, RF proved particularly effective, leveraging its ability to model nonlinear dependencies and extract robust patterns. Although LR yielded slightly lower accuracy, it exhibited strong generalization capabilities and served as a reliable baseline for comparison.

**Figure 12:**
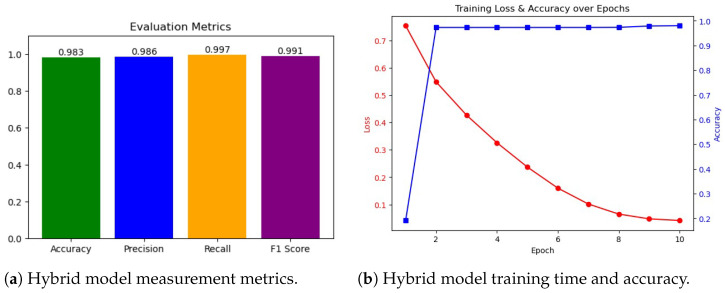
Experiment 1 results for the hybrid models. (**a**) Performance metrics (accuracy, precision, recall, and F1-Score). (**b**) Training and testing accuracy over epochs for the hybrid model.

As shown in Figure 12, the hybrid transformer–GCN model achieved 98.28% accuracy, with a precision of 98.59%, recall of 99.66%, and F1-score of 99.12%. While RF slightly outperformed the hybrid model with near-perfect results, the transformer–GCN model demonstrated superior robustness compared to LR and AdaBoost. Unlike tree-based ensembles, the hybrid model integrates graph-based representations with sequence learning, enabling it to capture temporal and structural dependencies in IoMT traffic. This makes it particularly promising for real-world anomaly detection, where unseen variations may challenge models optimized for binary tasks. Thus, the hybrid model provides a strong balance between accuracy and generalization.

### 5.2. Experiment 2 Results

**Figure 13:**
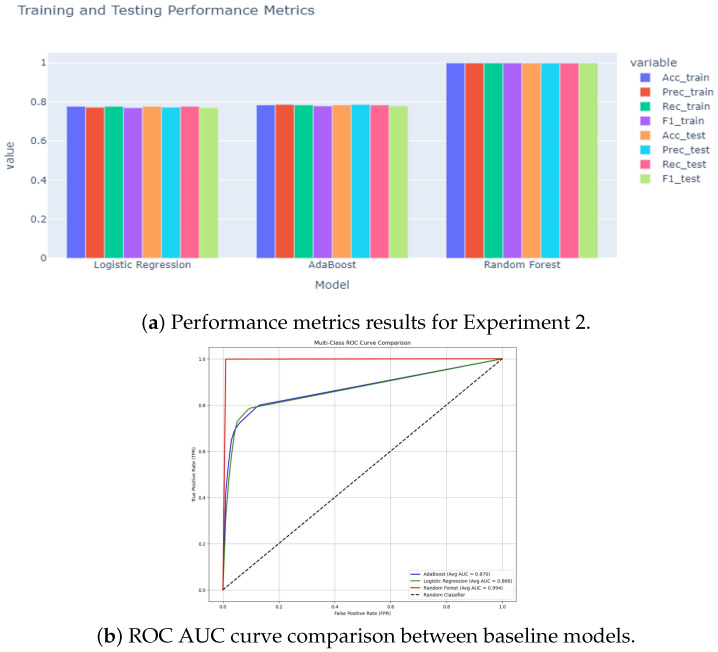
Experiment 2 results for the base models. (**a**) Accuracy, precision, recall, and F1-Score. (**b**) the ROC AUC curve comparison between baseline models.

**Figure 14:**
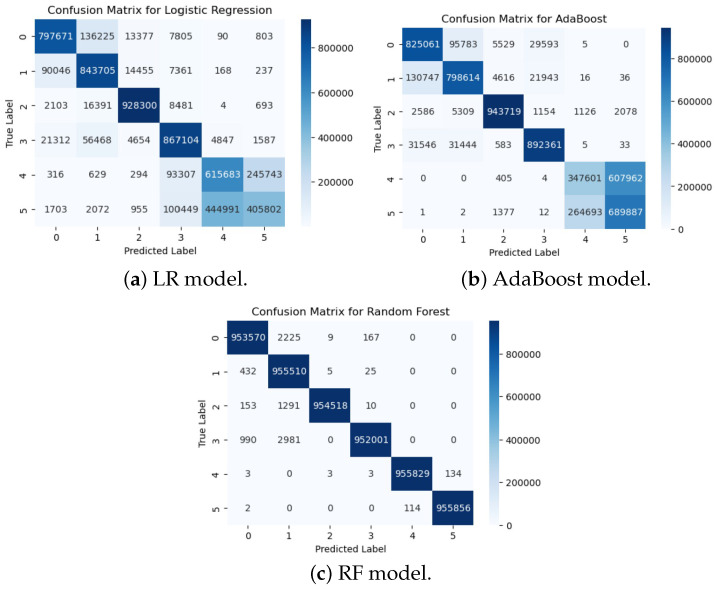
Confusion matrices for LR, AdaBoost, and RF models in Experiment 2.

In the second experiment, anomaly detection was performed over five anomaly classes and one benign class, as shown in Figure 13a, and the ROC AUC curve for the experiment is shown in Figure 13b. The confusion matrices for the second experiment are shown in Figure 14. The RF model again achieved the highest performance, with an accuracy of 0.9985, precision of 0.9985, recall of 0.9985, and F1-score of 0.9985, demonstrating its ability to effectively distinguish among multiple anomaly types. The AdaBoost classifier performed moderately well, achieving an accuracy of 0.7841, precision of 0.7866, recall of 0.7841, and F1-score of 0.7791. LR showed the lowest performance, with an accuracy of 0.7773, precision of 0.7725, recall of 0.7773, and F1-score of 0.7705, indicating that the linear model struggles to capture the more complex patterns required for multi-class anomaly detection. These results highlight that ensemble-based models, particularly RF, are highly effective for multi-class anomaly detection in IoMT environments, while simpler linear models provide a reasonable but lower-performing baseline.

**Figure 15:**
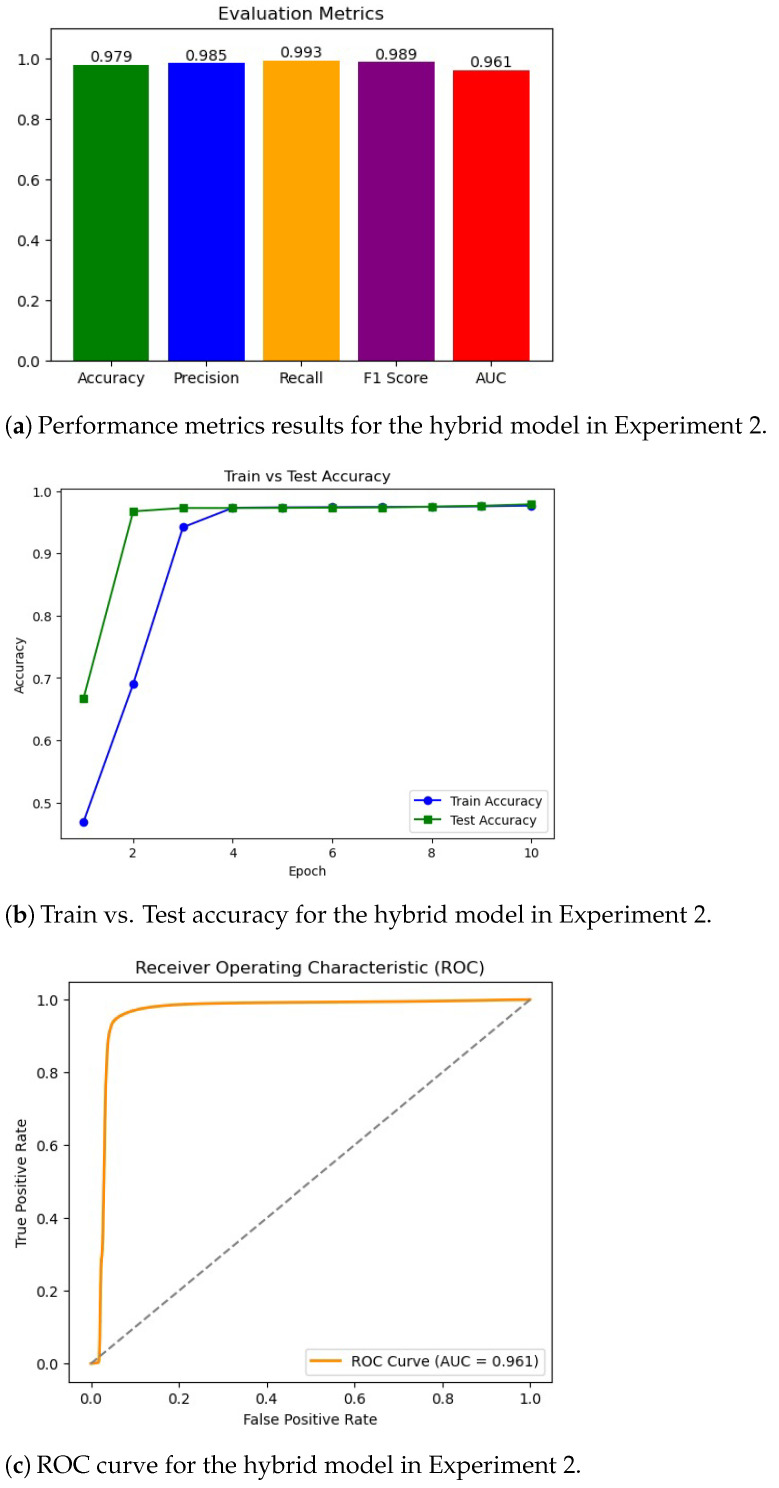
Experiment 2 results. (**a**) Performance metrics (accuracy, precision, recall, and F1-Score). (**b**) Training and testing accuracy over epochs. (**c**) ROC curve plot for the hybrid model.

As can be seen from Figure 15, the hybrid model achieved high performance, with a precision of 98.28%, a precision of 98.59%, a recall of 99.66%, and an F1-score of 99.12%. These results notably surpassed those of LR and AdaBoost, both of which failed to exceed 80% accuracy, and closely approached the optimal performance of RF. The hybrid model’s strength lies in its dual capacity to model structural dependencies through graph neural layers and capture temporal dynamics via transformer components. This architectural synergy enhances its adaptability to multi-class IoMT anomaly detection tasks, enabling effective handling of complex traffic patterns while maintaining strong generalization.

### 5.3. Experiment 3 Results

**Figure 16:**
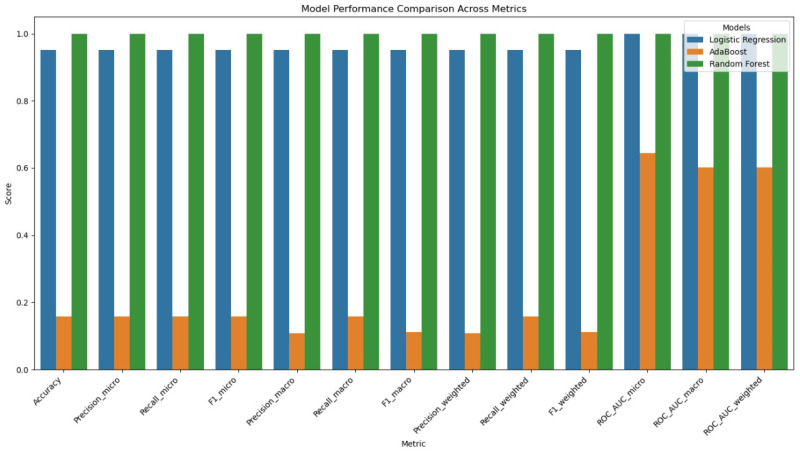
Performance metrics (accuracy, precision, recall, and F1-score) for the LR, AdaBoost, and RF models in Experiment 3. The LR and RF models achieved the highest performance, while the AdaBoost model performed the lowest.

**Figure 17:**
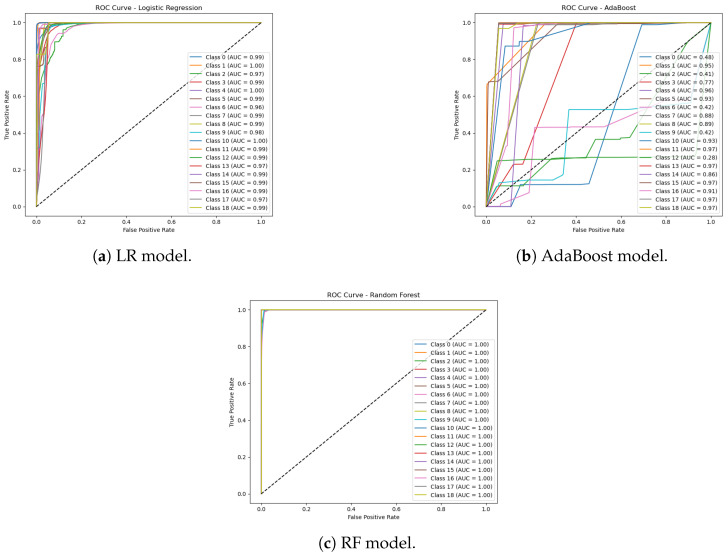
ROC AUC curve for LR, AdaBoost, and RF models run in Experiment 3.

**Figure 18:**
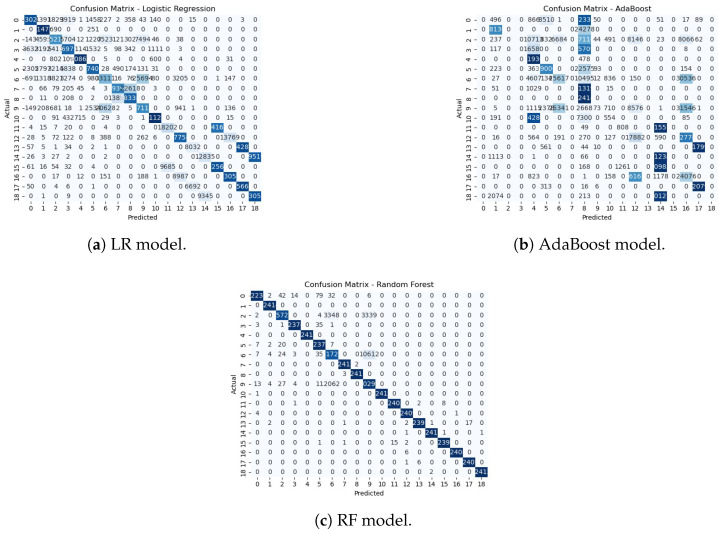
Confusion matrices for LR, AdaBoost, and RF models in Experiment 3.

In the third experimental phase, we assess the anomaly detection classification with challenging multi-class data that consist of 18 anomaly classes and one benign class, as shown in Figure 16. The ROC AUC curve for the experiment is shown in Figure 17. The confusion matrices for the first experiment are shown in Figure 18.

**Figure 19:**
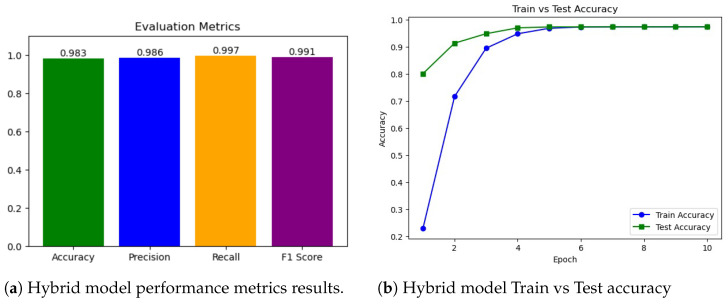
Experiment 3 results for the hybrid model. (**a**) Performance metrics (accuracy, precision, recall, and F1-Score). (**b**) Training and testing accuracy over epochs.

As shown in Figure 19, the transformer–GCN model achieved the best overall performance, with an accuracy of 98.28%, precision of 98.59%, F1-score of 99.12%, and an ROC AUC of 0.9980. These results highlight its ability to maintain robust classification even under the increased complexity of highly imbalanced and heterogeneous traffic patterns. Because the hybrid model combines structural dependencies through GCN layers with temporal and sequential modeling via the transformer layers, the model is able to generalize well to IoMT network traffic data with a diverse set of attack scenarios.

RF also delivered strong performance, with an accuracy of 98.10% and an F1-score of 98.59%. While slightly lower than the hybrid model, RF remains a highly competitive baseline. LR also performed strongly, with metrics near 0.951 and ROC AUC values exceeding 0.998, indicating robust generalization despite the increased categorical complexity. In contrast, AdaBoost struggled considerably, with performance collapsing to approximately 0.158 accuracy and ROC AUC values around 0.60–0.64, reflecting its limited adaptability when faced with a large number of anomaly classes.

## 6. Discussion

In the context of IoMT security, the CICIoMT2024 dataset offers several advantages. Firstly, a well-labeled and public IoMT dataset is a scarce resource that researchers worldwide should use to advance IoMT security and address real-world challenges in medical device security. Secondly, it serves as a comprehensive benchmark, addressing the limitations of existing IoMT datasets regarding device diversity, attack types, and profiling. Furthermore, it captures the lifecycle and behavioral patterns of IoMT devices, which includes critical functions for anomaly identification and the improvement of security solutions. The dataset also establishes a baseline for future research, supporting the development and evaluation of MML models for detecting and classifying IoMT cyberattacks. Lastly, it was tailored not only to general healthcare applications but also specifically focused on ensuring the confidentiality, integrity, and availability of safety-critical medical services.

In practical IoMT environments, achieving a low false-positive operating point is as critical as maximizing overall accuracy. Excessive false alarms can overload the monitoring system and desensitize operators, while undetected attacks may compromise patient safety. In our evaluation, the proposed GCN–transformer maintained a false-positive rate below 2% while sustaining a recall exceeding 98%. This indicates its ability to operate effectively in safety-critical domains where alarm precision is essential. Furthermore, inference latency averaged 42–55 ms per batch (depending on input window size), demonstrating that the model can support near real-time traffic analysis and early-stage anomaly detection within hospital networks.

However, the CICIoMT2024 dataset has certain limitations. The dataset’s evaluation is restricted to standard and lightweight ML models; more complex architectures could be designed to enhance classification performance. Although the dataset includes devices currently in use, IoMT technologies evolve rapidly, and future devices may introduce new challenges that will require updated datasets. Certain aspects of real-world deployability, such as real-time evaluation, efficiency, and the handling of zero-day attacks, are beyond the scope of this research. The dataset also relies on existing features extracted from PCAP files, but further feature engineering and optimization could improve detection capabilities. Additionally, combining this dataset with other healthcare datasets or simulation platforms could provide more comprehensive insights. These limitations highlight crucial areas for future research, including the development of advanced models, the incorporation of new devices, and the improvement of real-world applicability.

Notably, the RF model performs exceptionally well due to its ability to capture nonlinear feature interactions and hierarchical decision boundaries that align closely with the tabular and statistical nature of the CICIoMT2024 dataset. This suggests that the dataset’s features are well-structured and exhibit strong discriminative patterns, which are effectively exploited by tree-based ensemble methods. However, the proposed hybrid model demonstrates superior generalization and robustness, particularly in complex or noisy scenarios where temporal and relational dependencies become significant. The hybrid approach is thus more adaptable to dynamic IoT environments, where graph-based and sequence-based contextual learning can capture evolving attack behaviors not easily represented in fixed DT.

Transformer architectures present a distinct set of advantages and limitations. The primary strengths of transformers are their performance, efficiency, and flexibility. The self-attention mechanism enables the model to capture long-range dependencies more effectively than RNNs or CNNs, leading to state-of-the-art results in domains such as NLP, computer vision, and audio processing. Unlike sequential models, transformers process all input tokens in parallel, which significantly accelerates training and improves scalability for large datasets and models (e.g., BERT, GPT). This architecture provides a unified framework for various data types and is highly amenable to the pre-training and fine-tuning paradigm, reducing the data requirements for specific downstream tasks.

Despite these advantages, transformers have notable limitations. The self-attention mechanism has a computational and memory complexity of $O(T^{2})$ with respect to sequence length *T*, making it inefficient and resource-intensive for long sequences. Consequently, training large models requires specialized hardware (e.g., GPUs, TPUs). Lacking the inductive biases of RNNs and CNNs, transformers require extensive data to train effectively and are prone to overfitting on smaller datasets. Other challenges include the necessity of explicit positional encodings to incorporate sequence order, the poor interpretability of the attention mechanism, high latency during autoregressive inference, and the need for task-specific adaptations to achieve optimal performance [61].

## 7. Conclusions & Future Work

In this work, we introduced a novel hybrid anomaly detection model combining transformer–GCN layers to enhance security in IoMT environments. By leveraging GCNs to capture device relationships and transformers to model sequential dependencies, the approach achieved robust detection of both common and complex cyberattacks. Our evaluation on the large-scale CICIoMT24 dataset, simulating over 40 devices and 18 attack classes, confirmed the model’s effectiveness, consistently yielding high accuracy, precision, recall, and F1-scores across progressively challenging experiments.

While the RF model proved a competitive baseline in many settings—attesting to the strong tabular characteristics of the dataset—the hybrid model demonstrated superior scalability and generalization. Its ability to model relational and temporal dependencies makes it better suited for streaming IoT contexts and the detection of rare or low-frequency attacks where tree ensembles may fail. This study therefore confirms the potential of hybrid graph–sequence architectures as a scalable and generalizable solution for IoMT security, establishing the feasibility of the transformer–GCN architecture in this domain.

The successful real-world deployment of such models requires addressing critical operational risks, specifically the balance between false negatives (which compromise patient safety) and false positives (which cause alarm fatigue). To address this, the hybrid model was engineered to balance detection sensitivity, inference efficiency, and robustness, making it an essential tool for safe and sustainable AI-based anomaly detection in clinical settings.

Future work will focus on improving the model’s applicability by exploring richer graph construction strategies, implementing temporal graph attention mechanisms, and evaluating performance in real-time deployment scenarios to further enhance anomaly detection in IoMT systems.

## Figures and Tables

**Table 2:** Attack categories and counts in the CICIoMT24 dataset [15].

Class	Category	Attack	Count
Benign	-	-	192,732
Attack	Spoofing	ARP Spoofing	17,791
	Recon	VulScan	3207
		OS Scan	20,666
		Port Scan	106,603
		Ping Sweep	926
	DDoS	TCP	462,480
		ICMP	514,724
		SYN	540,498
		UDP	704,503
	DoS	DoS SYN	974,359
		DoS TCP	987,063
		DoS ICMP	1,887,175
		DoS UDP	1,998,026
	MQTT	DDoS Connect Flood	214,952
		DoS Publish Flood	52,881
		DoS Connect Flood	15,904
		Malformed Data	6877
		DDoS Publish Flood	36,039

**Table 3:** Hyperparameters for all models used in the experiments.

Model	Hyperparameter	Value
LR	penalty	‘l2’
	dual	False
	tol	0.0001
	C	1.0
	fit_intercept	True
	solver	‘lbfgs’
	max_iter	100
	multi_class	‘auto’
	random_state	None
AdaBoost	base_estimator	DecisionTreeClassifier
	n_estimators	50
	learning_rate	1.0
	algorithm	‘SAMME.R’
	random_state	None
RF	n_estimators	100
	criterion	‘gini’
	max_depth	None
	min_samples_split	2
	min_samples_leaf	1
	max_features	‘sqrt’
	bootstrap	True
	oob_score	False
	random_state	None
Hybrid Model	proj_dim	48
	n_heads	4
	num_layers	2
	hidden_dim	64
	dim_feedforward	64
	d_model	48
	learning_rate	0.001
	epochs	10
	optimizer	Adam

**Table 4:** Comparison of LR, AdaBoost, and RF models, and the proposed hybrid GNN+transformer model across all three experiments, where the best results are highlighted in **bold** font.

Experiment	Model	Accuracy	Precision	Recall	F1-Score	ROC AUC
Exp. 1 (Binary)	LR	0.9921	0.9969	0.9872	0.9920	0.9979
	AdaBoost	0.9988	0.9998	0.9978	0.9988	0.9999
	RF	**0.9996**	**0.9917**	**0.9992**	**0.9996**	**1.0000**
	Transformer Only	0.99	0.995	0.919	0.9938	0.9956
	GCN Only	0.9981	0.978	0.985	0.98	0.99
	Transformer–GCN	0.983	0.986	0.9966	0.9912	0.9980
Exp. 2 (6 Classes)	LR	0.7773	0.7725	0.7773	0.7705	0.8730
	AdaBoost	0.7841	0.7866	0.7841	0.7791	0.8890
	RF	**0.9985**	**0.9985**	0.9985	0.9985	0.9902
	Transformer Only	0.98	0.9933	0.99	**0.9988**	**0.9915**
	GCN Only	0.9751	0.9782	**1**	0.9864	0.9822
	Transformer–GCN	0.979	0.985	0.993	0.989	0.961
Exp. 3 (19 Classes)	LR	0.9512	0.9515	0.9512	0.9512	0.9986
	AdaBoost	0.1579	0.1084	0.1579	0.1111	0.6023
	RF	0.9810	0.9802	0.9858	0.9859	0.9898
	Transformer–GCN	**0.9828**	**0.9859**	0.9966	**0.9912**	**0.9980**

## Data Availability

The dataset is available at https://www.unb.ca/cic/datasets/iomt-dataset-2024.html, accessed on 30 November 2024.

## Abbreviations

The following abbreviations are used in this manuscript:

AdaBoost	Adaptive Boosting
GCN	Graph Convolutional Network
IoMT	Internet of Medical Things
LR	Logistic Regression
ML	Machine Learning
RF	Random Forest
